# Tear lactate improves the evaluation of proliferative diabetic retinopathy in type-2 diabetes patients

**DOI:** 10.1186/s43556-025-00297-0

**Published:** 2025-07-18

**Authors:** Xin Wen, Tsz Kin Ng, Guihua Zhang, Haoyu Chen, Zhenggen Wu, Qingping Liu, Mingzhi Zhang

**Affiliations:** 1 Joint Shantou International Eye Center of Shantou University and the Chinese University of Hong Kong, Shantou, Guangdong, China; 2 Shantou University Medical College, Shantou, Guangdong China; 3 Shantou University Medical College Eye Research Institute, Shantou, Guangdong China; 4 Department of Ophthalmology and Visual Sciences, The Chinese University of Hong Kong, Hong Kong, China

**Keywords:** Diabetic retinopathy, Tears, Marker, Lactate, Monosaccharides

## Abstract

Proliferative diabetic retinopathy (PDR) is the advanced stage of DR and characterized by retinal neovascularization (RNV). The diagnosis of PDR relies primarily on imaging features and blood glucose levels. Whether early biomarkers in other biofluid applied in the evaluation of PDR and RNV remain elusive. In total, 40 Chinese type-2 diabetes with DR and 21 non-diabetic subjects were recruited. Tear glycometabolic profiles and glycometabolite levels were comprehensively analyzed using both untargeted and targeted metabolomics approaches. Additionally, we employed multivariable logistic regression models, Pearson correlation analysis, receiver operating characteristic curve (ROC), retinal non-perfusion area detection and choroid sprouting assay to evaluate and validate the association between tear metabolites and PDR. Our metabolomic analysis revealed significantly elevated levels of metabolites related to the TCA cycle as well as D-glutamine and D-glutamate pathway in PDR subjects compared to non-diabetic controls. Among these metabolites, the fasting tear lactate was the highest in PDR subjects relative to other tear monosaccharides. Notably, tear lactate emerged as an independent risk factor for PDR, achieving an area under the curve (AUC) of 0.896 in predictive modeling. Furthermore, the tear lactate was validated to have effect on RNV. In summary, the study delineated glycometabolic features in tears of type-2 diabetes patients with PDR and identified tear lactate could be a promising novel marker for PDR evaluation.

**Supplementary Information:**

The online version contains supplementary material available at10.1186/s43556-025-00297-0.

## Introduction

Diabetic retinopathy (DR), a microvascular ocular complication of diabetes mellitus (DM), is a leading cause of irreversible blindness and visual impairment among the working-age individuals [1]. In 2020, the number of adults with DR was estimated at 103.1 million worldwide, and it is expected to increase to 160.5 million by 2045 [2]. The global prevalence of DR was estimated to be 22.27% within the DM population. DR progressed gradually from non-proliferative DR (NPDR) to proliferative DR (PDR), which is the most vision-threatening manifestation of DR and characterized by retinal neovascularization (RNV) [3, 4]. The late-stage DR creates a considerable economic burden globally and causes significant psychological and emotional strains [5, 6]. Although fundus fluorescein angiography (FFA) and blood glucose analysis offer accurate diagnosis of PDR[7, 8], their invasiveness and inability to predict pre-lesional stages remain major drawbacks. Early identification of DR or its advanced forms before clinical onset may enable timely intervention, preventing further microvascular damage and severe vision loss. Since there is still lack of definite markers for the prediction of DR severity, searching for additional indicator to facilitate PDR evaluation is warranted.

The metabolic disturbances associated with DR manifest prior to observable clinical symptoms [9]. The metabolites and pathways associated with monosaccharides (glucose, fructose, galactose, mannose) and lactate play crucial roles in DR [10]. A previous metabolomics study found that significant alterations in glycolysis/gluconeogenesis and galactose metabolism pathways in the aqueous humor of DR patients compared to non-diabetic controls [11]. Moreover, the glycolysis metabolism, TCA metabolism, urea cycle metabolism, polyol metabolism were mainly associated with DR-induced metabolic disturbance in serum samples [12]. Additionally, proteomic analysis of vitreous exosomes in PDR individuals indicated that the differential proteins were primarily involved in glycolysis/gluconeogenesis, fructose and mannose metabolism [13]. Although the analysis of vitreous humor, aqueous humor, and serum may provide critical insights into ocular metabolic alterations in DR patients, these invasive sampling techniques pose inherent limitations for routine clinical implementation. The collection of intraocular fluids entails surgical intervention with associated risks of iatrogenic complications and sample contamination, while peripheral blood sampling introduces systemic confounding factors that may compromise metabolic interpretation [14].

Non-invasive monitoring of biochemical markers has gained significant attention, as molecular profiling of biofluids can potentially delineate both disease progression and underlying pathophysiological states [15]. As an accessible ocular biofluid, tears exhibit unique advantages in ophthalmic diagnostics due to their non-invasive collectability. Serving as dynamic biomarker reservoirs, they concurrently reflect anterior segment pathologies while offering molecular insights into posterior segment disorders [16, 17]. In DR patients, hyperglycemia can break the tight junction of pericyte and increase intercellular permeability so that glucose in the posterior eye segment could enter anterior eye segment though plasma leakage [18, 19]. It has been verified that tear glucose level is positively correlated with the blood glucose level in DM patients [20–22]. Therefore, tear glucose levels may represent alterations in ocular metabolism in DR patients.

Here, we aimed to characterize the glycometabolic profile in tears from type 2 DM patients with DR using metabolomics approach. The levels of tear metabolites were quantified and their association with DR severity were analyzed through correlation analysis and logistic regression model. Additionally, their predictive performances differentiating PDR from NPDR were evaluated. Furthermore, we validated the impact of tear metabolites on RNV by the retinal non-perfusion area of FFA and choroidal sprouting assays.

## Results

### Clinical characteristics in proliferative diabetic retinopathy subjects diverge from non-proliferative diabetic retinopathy and non-diabetic subjects

To systematically evaluate the characteristics of our cohort, we analyzed different types of clinical parameters of the enrolled participants (Table 1**)**. Some of the patients and data in control and NPDR group were cited from our previously published study [16]. In brief, for the demographics features, the mean age of the PDR subjects (58.63 ± 8.11 years) was significantly lower than that of the non-diabetic (68.10 ± 7.46 years, *P* < 0.001) and NPDR subjects (64.71 ± 6.13 years, *P* = 0.027). There were more female subjects in the PDR group (79.0%) than the non-diabetic group (42.9%, *P* = 0.027). For the blood biochemical indicators, the fasting blood glucose levels of PDR (9.79 ± 4.24 mM, *P* < 0.001) and NPDR subjects (8.68 ± 2.56 mM, *P* = 0.012) were significantly higher than that of the non-diabetic subjects (6.10 ± 0.61 mM). In addition, the HDL level of the PDR patients (1.26 ± 0.19 mM) was significantly lower than that of NPDR subjects (1.61 ± 1.48 mM, *P* = 0.025). Other clinical parameters also showed no statistically significant differences among the three study groups. For the ophthalmic features, the incidence of diabetic macula edema (DME) in PDR group (79.0%) was significantly higher than that in NPDR group (38.1%, *P* = 0.012). However, the percentage of laser treatment in PDR group (63.2%) showed no statistically significant differences in NPDR group (28.6%, *P* = 0.055). Therefore, we revealed PDR subjects exhibit distinct clinical characteristics that differentiate from both NPDR and non-diabetic cohorts.

**Table 1:** Demographics and clinical measurements of the study subjects

Characteristics	Control (*n* = 21)	NPDR (*n* = 21)	PDR (*n* = 19)	*P* (NPDR vs Control)	*P* (PDR vs Control)	*P* (PDR vs NPDR)
**Demographics features**						
Age (years; ± SD)	68.10 ± 7.46	64.71 ± 6.13	58.63 ± 8.11	0.293	** < 0.001**	**0.027**
Sex (n; %)						
Female	9 (42.86)	11 (52.38)	15 (78.95)	0.537*	0.027*	0.105*
Male	12 (57.14)	10 (47.62)	4 (22.05)			
DM Duration (years; ± SD)	N/A	7.00 ± 4.53	7.89 ± 3.94	/	/	0.332
DR Duration (years; ± SD)	N/A	1.42 ± 1.32	1.41 ± 1.27	/	/	0.984
**Biochemistry features**						
Blood glucose (mM)	6.10 ± 0.61	8.68 ± 2.56	9.79 ± 4.24	**0.012**	** < 0.001**	0.388
HbA1c (%)	N/A	8.13 ± 1.64	8.42 ± 1.63	/	/	0.877
Fructosamine (mM; ± SD)	0.27 ± 0.03	0.30 ± 0.05	0.31 ± 0.05	0.062	0.071	0.971
TG (mM; ± SD)	2.08 ± 2.19	1.54 ± 0.82	3.45 ± 6.87	0.946	0.599	0.449
TC (mM; ± SD)	5.56 ± 1.04	5.33 ± 0.90	6.39 ± 2.46	0.929	0.354	0.227
HDL (mM; ± SD)	1.47 ± 0.38	1.61 ± 0.48	1.26 ± 0.19	0.567	0.152	**0.025**
LDL (mM; ± SD)	3.36 ± 0.90	3.13 ± 0.96	3.76 ± 1.51	0.854	0.653	0.391
Urea (mM; ± SD)	6.03 ± 1.80	7.59 ± 3.91	10.49 ± 9.19	0.730	0.121	0.391
Creatinine (mM; ± SD)	77.11 ± 15.18	146.56 ± 199.37	126.39 ± 124.88	0.279	0.596	0.890
**Ophthalmic features**						
DME (n; %)						
Yes	0 (0)	8 (38.10)	15 (78.95)	**0.003**	** < 0.001**	**0.012**
No	21 (100)	13 (61.90)	4 (21.05)			
Laser treatment (n; %)						
Yes	0 (0)	6 (28.57)	12 (63.16)	**0.021**	** < 0.001**	0.055
No	21 (100)	15 (71.43)	7 (36.84)			

Some of the patients and data were cited from our previously published study [16]

DM diabetes mellitus, DR diabetic retinopathy, TG Triglycerides, TC Total cholesterol, HDL high density lipoprotein, LDL low density lipoprotein, DME diabetic macula edema

^*^Fisher’s exact test

### D-glutamine/glutamate metabolism and citrate acid cycle show significant activation in proliferative diabetic retinopathy cohort

**Fig. 1:**
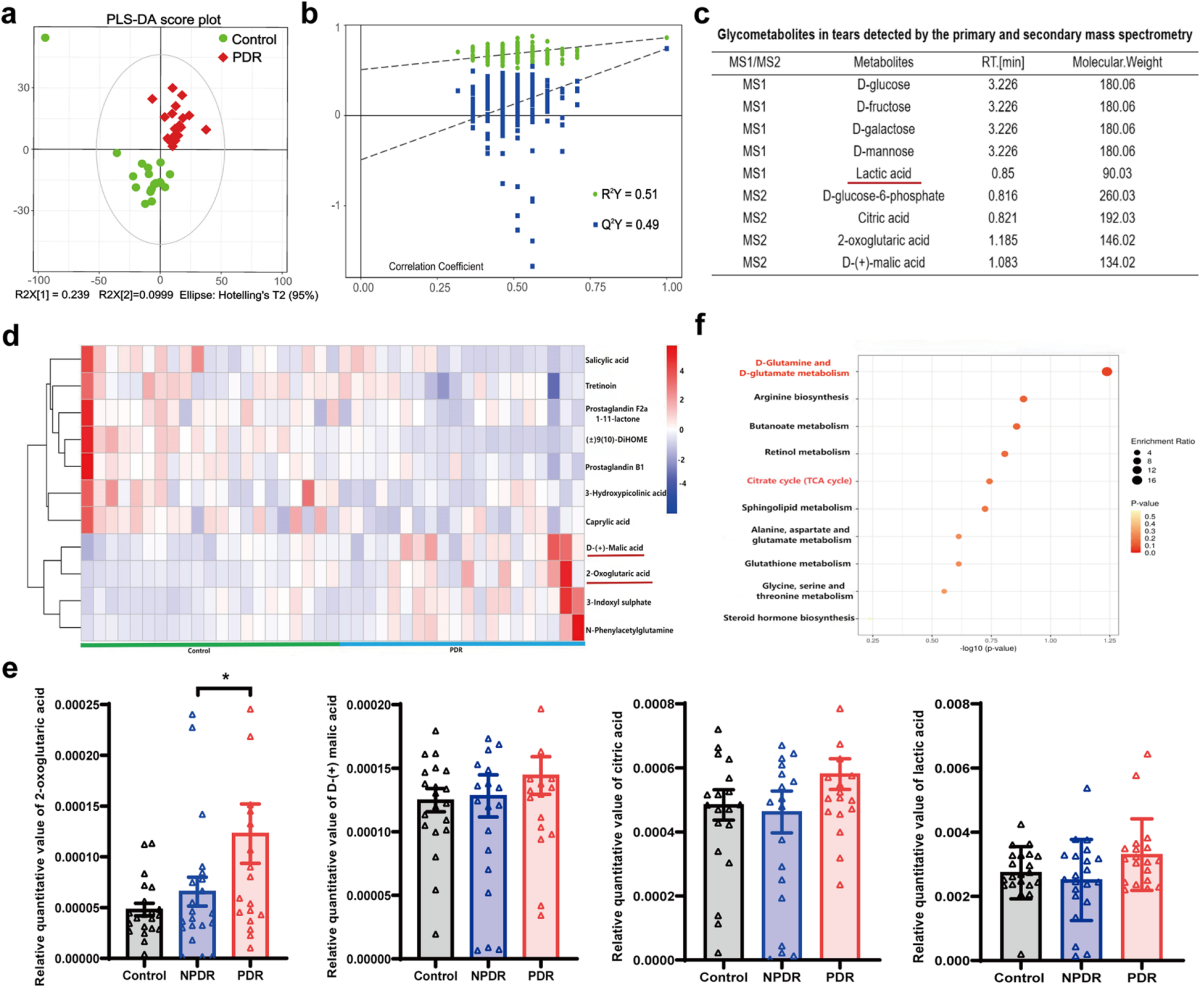
Untargeted metabolomics analysis of DR subjects in tear samples among the diabetic retinopathy and non-diabetic control subjects. (**a, b**) The score plot of PLS-DA and the permutation test analysis in control (n = 21) vs PDR group (n = 19); (**c**) Glycometabolites in tear samples detected by the primary and secondary mass spectrometry; (**d**) Heatmaps of significantly differentially abundant glycometabolites of PDR vs non-diabetic group; (**e**) The relative quantitative values of glycometabolites related to TCA cycle (control group: n = 21; NPDR group: n = 21; PDR group: n = 19); (**f**) D-glutamine and D-glutamate metabolism and TCA cycle were significantly highlighted in the comparison between the PDR and non-diabetic groups. NPDR: non-proliferative diabetic retinopathy; PDR: proliferative diabetic retinopathy

To investigate glycometabolic features in tear samples of PDR subjects, untargeted glycometabolomics analysis was carried out. There was a clear separation in the tear metabolic profiles between the control and PDR groups based on the PLS-DA models (Fig. 1a). The permutation test indicated that the models were reliable, with an R^2^Y of 0.51 and a Q^2^Y of 0.49 for the comparison between PDR and control groups (Fig. 1b). The LC/MS platform detected several metabolites related to glycometabolism in tears by the primary and secondary mass spectrometry qualitative matching analysis (Fig. 1c). Among them, 2-oxoglutaric acid and D-(+)-malic acid were significantly differential abundant glycometabolites between PDR and non-diabetic groups (Fig. 1d). The relative quantitative values of 2-oxoglutaric acid, D-(+)-malic acid, citric acid and lactic acid were higher in PDR subjects than in the controls (Fig. 1e). Furthermore, D-glutamine and D-glutamate metabolism and citrate acid cycle (TCA cycle) was found to be significantly highlighted in the comparison between the PDR and non-diabetic groups (Fig. 1f). These results reflected the glycometabolic characteristics and trend in tears of PDR subjects.

### Markedly elevation of tear lactate and monosaccharides levels of the proliferative diabetic retinopathy subjects

**Fig. 2:**
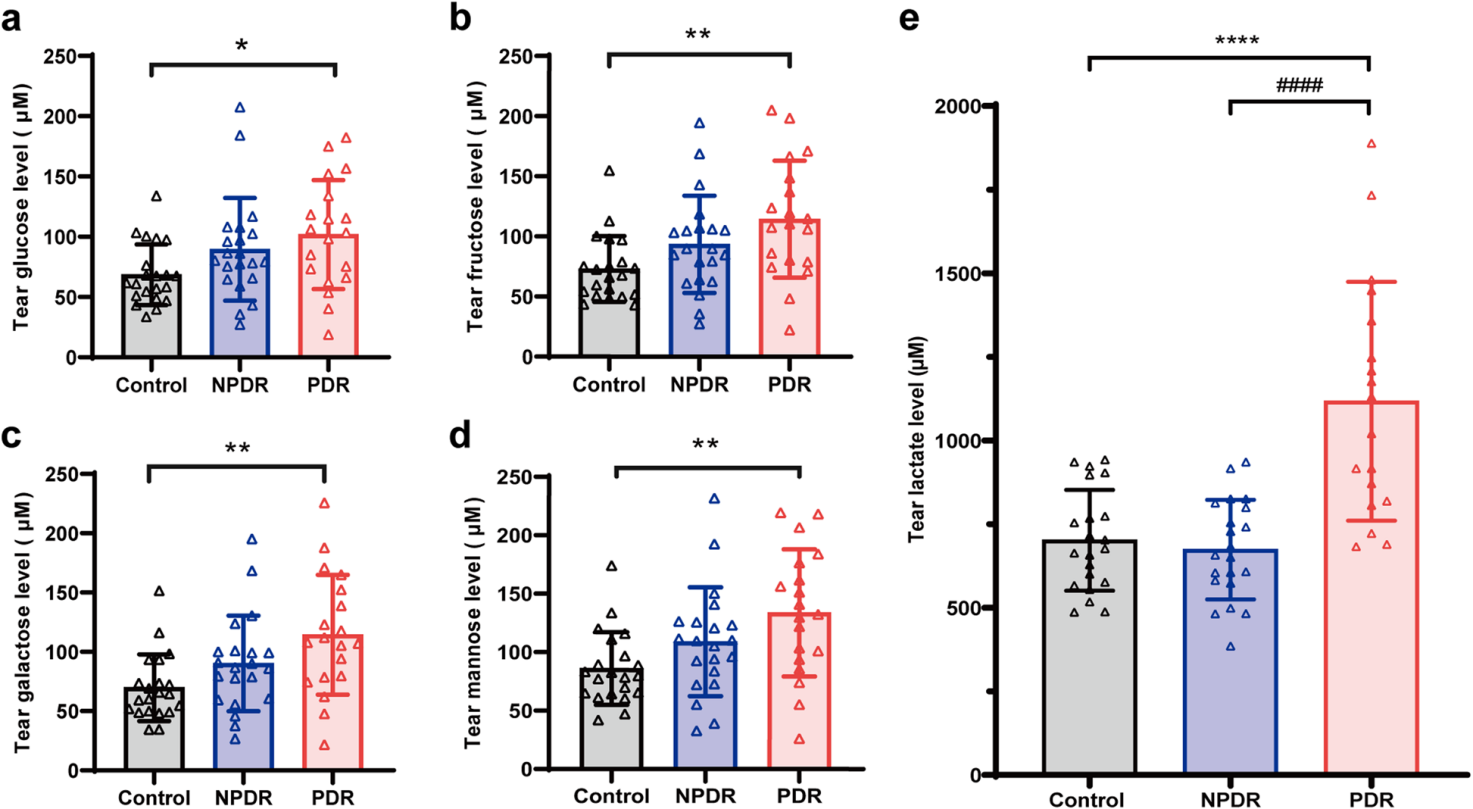
Targeted metabolomics analysis about the levels of lactate and monosaccharides (glucose, fructose, galactose, mannose) in tear samples among the diabetic retinopathy (n = 40) and non-diabetic control subjects (n = 21). (**a**) Tear glucose; (**b**) Tear fructose; (**c**) Tear galactose; (**d**) Tear mannose; **(e)** Tear lactate; *P < 0.05, **P < 0.01, ****P < 0.0001 as compared to the control subjects; #### *P* < 0.0001 as compared to the non-proliferative diabetic retinopathy (NPDR) subjects. PDR: proliferative diabetic retinopathy

To further quantify the levels of glycometabolites in the primary mass spectrometry (Lactate, D-glucose, D-fructose, D-galactose and D-mannose) in tear samples of DR subjects, targeted glycometabolomics analysis was performed. The results showed that the levels of tear glucose (101.73 ± 45.18 μM, *P* = 0.023; Fig. 2a), fructose (114.30 ± 38.67 μM, *P* = 0.004; Fig. 2b), galactose (114.34 ± 50.58 μM, *P* = 0.003; Fig. 2c), mannose (133.69 ± 54.35 μM, *P* = 0.004; Fig. 2d), and lactate (1212.09 ± 538.65 μM, *P* < 0.001; Fig. 2e) of the PDR subjects at fasting were significantly higher than those of the non-diabetic subjects. In contrast, there were no statistically significant differences in the levels of tear monosaccharides (D-glucose, D-fructose, D-galactose, D-mannose) and lactate in the tear samples of the NPDR subjects at fasting as compared to the non-diabetic subjects. Moreover, the tear lactate level of the PDR subjects (1212.09 ± 538.65 μM) was also significantly higher than that of the NPDR subjects (674.09 ± 148.86 μM, *P* < 0.001). These results revealed that the fasting tear lactate levels were markedly higher in PDR subjects compared to other monosaccharides.

### The tear lactate level is an independent risk factor for proliferative diabetic retinopathy subjects

**Fig. 3:**
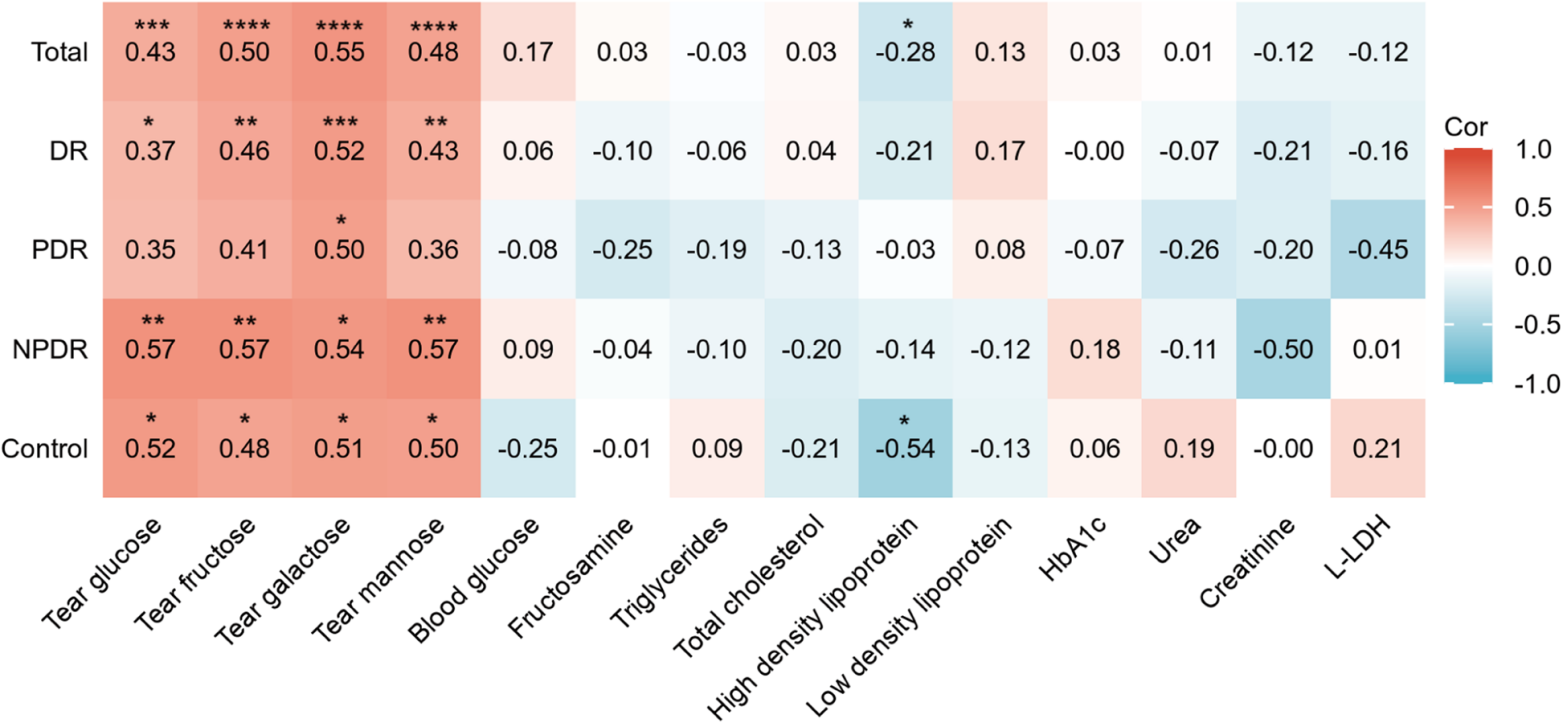
Correlation analysis for the tear lactate level with tear monosaccharides levels or blood biochemical indicators levels among the diabetic retinopathy (n = 40) and non-diabetic control subjects (n = 21). Weak or moderate correlations were found for the tear lactate level with tear monosaccharides. No correlations were found for the tear lactate level with blood biochemical indicators levels. (*P < 0.05, **P < 0.01, ***P < 0.001, ****P < 0.0001)

To evaluate the relationship between tear or blood monosaccharides levels and PDR status, multivariable logistic regression models were fitted. The results indicated that elevated tear lactate level is a significant risk for PDR (OR: 1.010, 95% C.I.: 1.003–1.016; *P* = 0.005; Model 1, Table 2). A similar result was obtained after adjusting demographic and DR durations (OR: 1.016, 95% C.I.: 1.003–1.029; *P* = 0.012; Model 2, Table 2), which means the risk of PDR increased 1.016 times for every 1 μM increment in tear lactate level. In contrast, none of the other tear metabolites (glucose, fructose, galactose, or mannose) or blood glucose reached statistical significance in multivariable logistic regression models (*P* > 0.05, Table 2). To further examine the association of tear lactate with other tear metabolites or blood biochemical indicators, correlation analysis was performed. Significantly weak or moderate correlations were found for the tear lactate level with tear monosaccharides for DR subjects and its subgroups (Pearson *r* = 0.35–0.57, *P* < 0.05, Fig. 3). On the contrary, no correlations were found for the tear lactate level with blood biochemical indicators levels, especially the blood glucose and HbA1c levels (Fig. 3). Altogether, these results indicated that the tear lactate level is a risk factor for PDR subjects and regulated independently of blood glucose level.

**Table 2:** Association of metabolites with proliferative diabetic retinopathy in multivariable logistic regression models

Independent variable	Model 1		Model 2	
	OR (95% CI)	*P* value	OR (95% CI)	*P* value
Tear lactate	1.010 (1.003–1.016)	0.005	1.016 (1.003–1.029)	0.012
Tear glucose	1.007 (0.992–1.022)	0.377	1.009 (0.990–1.028)	0.347
Tear fructose	1.011 (0.996–1.026)	0.150	1.016 (0.995–1.036)	0.130
Tear galactose	1.012 (0.997–1.028)	0.110	1.018 (0.997–1.040)	0.130
Tear mannose	1.010 (0.997–1.024)	0.133	1.015 (0.997–1.033)	0.112
Blood glucose	1.104 (0.909–1.342)	0.319	1.133 (0.881–1.458)	0.330

Logistic regression: dependent variable, proliferative diabetic retinopathy (yes or no); independent variable, tear lactate, tear glucose, tear fructose, tear galactose, tear mannose, blood glucose

Model 1: Logistic regression of tear lactate, tear glucose, tear fructose, tear galactose, tear mannose, blood glucose, respectively

Model 2: Adjusted age, sex, DM duration and DR duration above model 1

### Tear lactate levels perform better to predict PDR than blood glucose and other monosaccharide levels

**Fig. 4:**
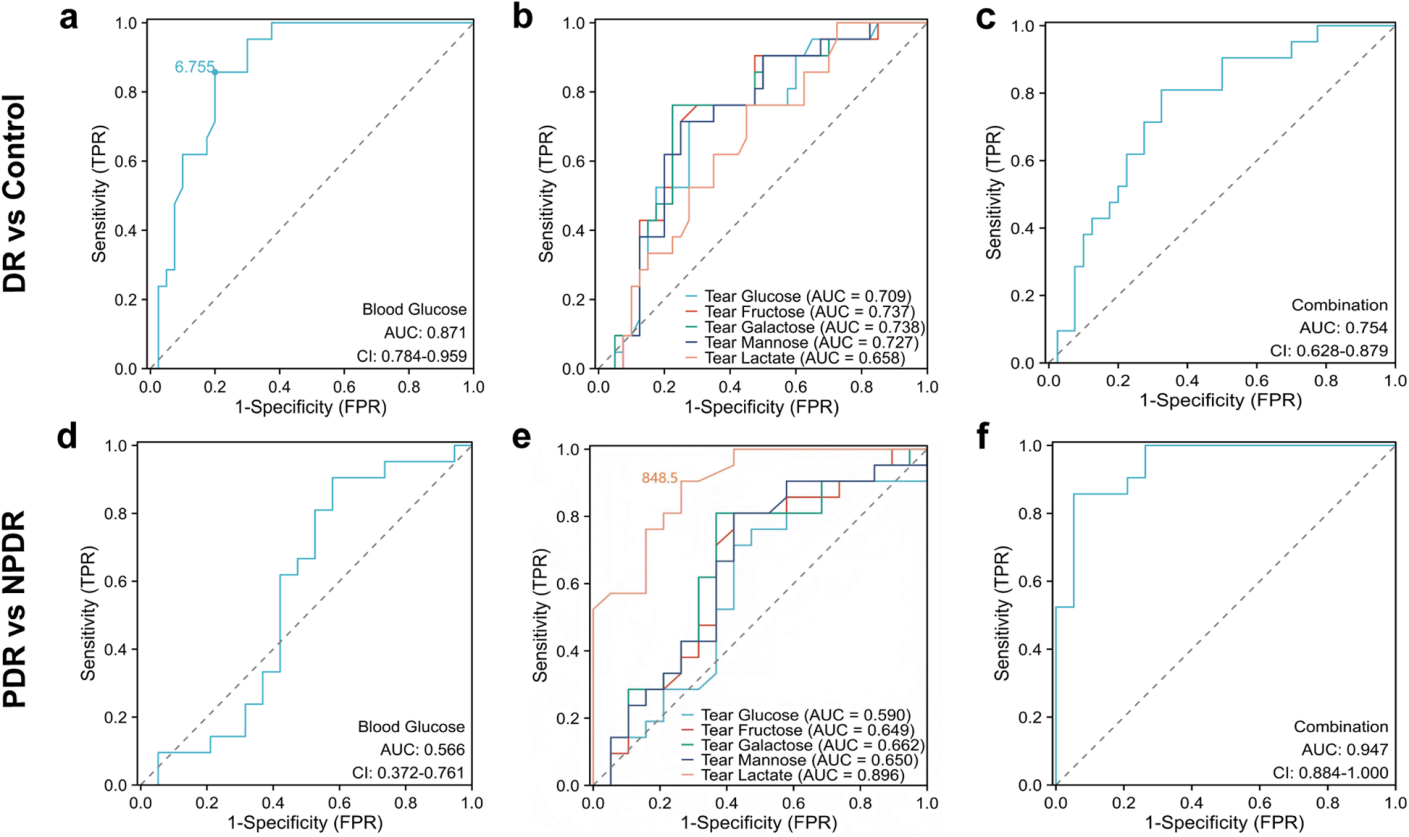
Receiver operating characteristic analysis on the fasting blood glucose and tear monosaccharides and lactate levels. **(a-c)** The comparison between the diabetic retinopathy (DR) (n = 40) and non-diabetic control subjects (n = 21); **(d-f)** The comparison between the proliferative DR (PDR) (n = 19) and non-proliferative DR (NPDR) subjects (n = 21); **(a and d)** The receiver operating characteristic (ROC) analysis on the fasting blood glucose level; **(b and e)** The ROC analysis on the fasting tear glucose, fructose, galactose, mannose, and lactate levels; **(c and f)** The ROC analysis on the combined tear glucose, fructose, galactose, mannose, and lactate levels. AUC: the area under ROC curve. CI: 95% confident intervals

To evaluate the predictive performance of tear lactate and monosaccharides levels, the ROC analysis was performed (Table S1). For the comparison between the DR subjects and the non-diabetic control subjects, the fasting blood glucose level achieved an area under ROC curve (AUC) of 0.871 (95% confident intervals (C.I.): 0.784 ‒ 0.959; Fig. 4a) and the cut-off value of the blood glucose to distinguish DR and control patients is 6.755 mM. In contrast, tear glucose, fructose, galactose, mannose, and lactate achieved an AUC of 0.709 (95% C.I.: 0.574 ‒ 0.844), 0.737 (95% C.I.: 0.607 ‒ 0.867), 0.738 (95% C.I.: 0.607 ‒ 0.869), 0.727 (95% C.I.: 0.596 ‒ 0.859), and 0.658 (95% C.I.: 0.519 ‒ 0.797) respectively (Fig. 4b). The combination of 4 monosaccharides and lactate in tears achieved an AUC of 0.754 (95% C.I.: 0.628 ‒ 0.879) for the comparison between the DR and non-diabetic subjects (Fig. 4c). For the comparison between the PDR and NPDR subjects, the fasting blood glucose level achieved an AUC of 0.566 (95% C.I.: 0.372 ‒ 0.761; Fig. 4d). On the contrary, tear glucose, fructose, galactose, mannose, and lactate achieved an AUC of 0.590 (95% C.I.: 0.403 ‒ 0.777), 0.649 (95% C.I.: 0.470 ‒ 0.828), 0.662 (95% C.I.: 0.484 ‒ 0.839), 0.650 (95% C.I.: 0.471 ‒ 0.830), and 0.896 (95% C.I.: 0.802 ‒ 0.990) respectively (Fig. 4e). The cut-off value of the tear lactate to distinguish NPDR and PDR is 848.5 μM. The combination of 4 monosaccharides and lactate in tears achieved an AUC of 0.947 (95% C.I.: 0.884 ‒ 1.000) for the comparison between the PDR and NPDR subjects (Fig. 4f). The results demonstrated that the tear lactate levels perform better to predict PDR status than the blood glucose level and other monosaccharide levels.

### Validation of the effect of tear lactate on retinal neovascularization

**Fig. 5:**
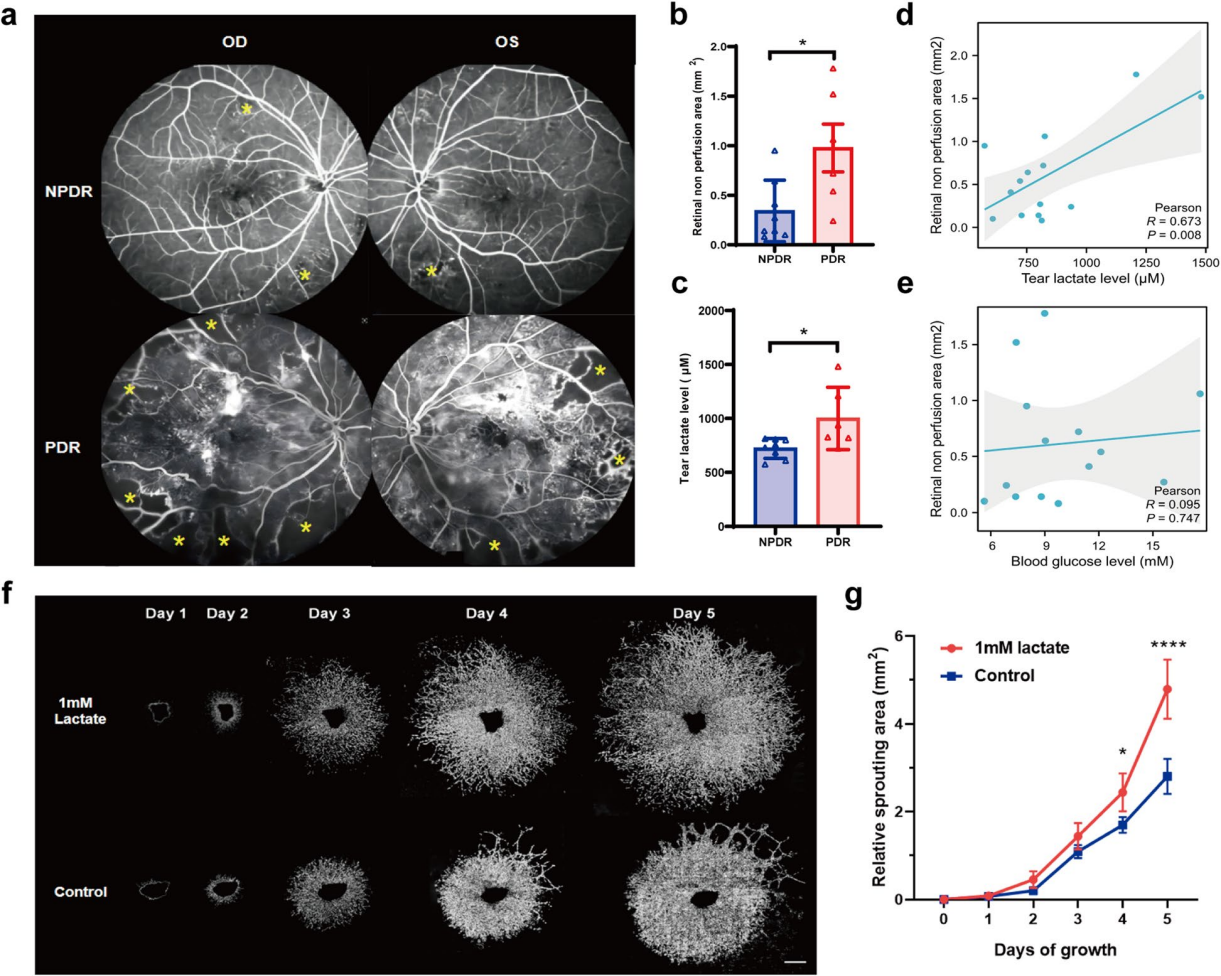
Evaluation of the tear lactate level on retinal neovascularization.** (a)** FFA images of non-perfusion area of retinal posterior pole from NPDR (n = 8) and PDR subjects (n = 6) (*: retinal non perfusion area); **(b)** The retinal non perfusion area in PDR subjects was larger than that in NPDR subjects;** (c)** The tear lactate level in PDR subjects was higher than that in NPDR subjects; **(d)** Correlations of tear lactate level with retinal non perfusion area (n = 14). **(e)** Correlations of blood glucose level with retinal non perfusion area;** (f-g)** Images and statistical analysis of relative sprouting area from lactate and control groups (Scale bar: 500 μm). OD: Oculus Dexter; OS: Oculus Sinister

To validate the effect of the tear lactate and blood glucose level on retinal neovascularization, the retinal non-perfusion areas of FFA was examined. FFA examinations were conducted for the NPDR and PDR subjects who did not receive retinal laser photocoagulation therapy (Fig. 5a). The average retinal non-perfusion area in posterior pole from PDR subjects (0.98 ± 0.59 mm^2^) was significantly larger than that from NPDR subjects (0.34 ± 0.30 mm^2^, *P* = 0.0225, Fig. 5b). Meanwhile, the tear lactate level in PDR subjects (998.7 ± 289.4 μM) was significantly higher than that in NPDR subjects (720.8 ± 91.85 μM, *P* = 0.0242; Fig. 5c). Furthermore, significant correlation was found for the tear lactate levels with the retinal non-perfusion areas (r = 0.673, *P* = 0.008, Fig. 5d). In contrast, no significant correlation was observed for the blood glucose levels with the retinal non-perfusion areas (r = 0.095, *P* = 0.747, Fig. 5e). Subsequently, to eliminate the interference of hyperglycemia to RNV, choroid sprouting assay of non-diabetic mice was further carried out (Fig. 5f). The sprouting area of experimental group (1 mM lactate) in Day4 (2.440 ± 0.431 mm^2^ vs 1.698 ± 0.679 mm^2^, *P* = 0.0424) and Day5 (4.790 ± 0.672 vs 2.805 ± 0.397, *P* < 0.0001) was significantly larger than that in control group. The results further validated the effect of tear lactate on RNV.

## Discussion

**Fig. 6:**
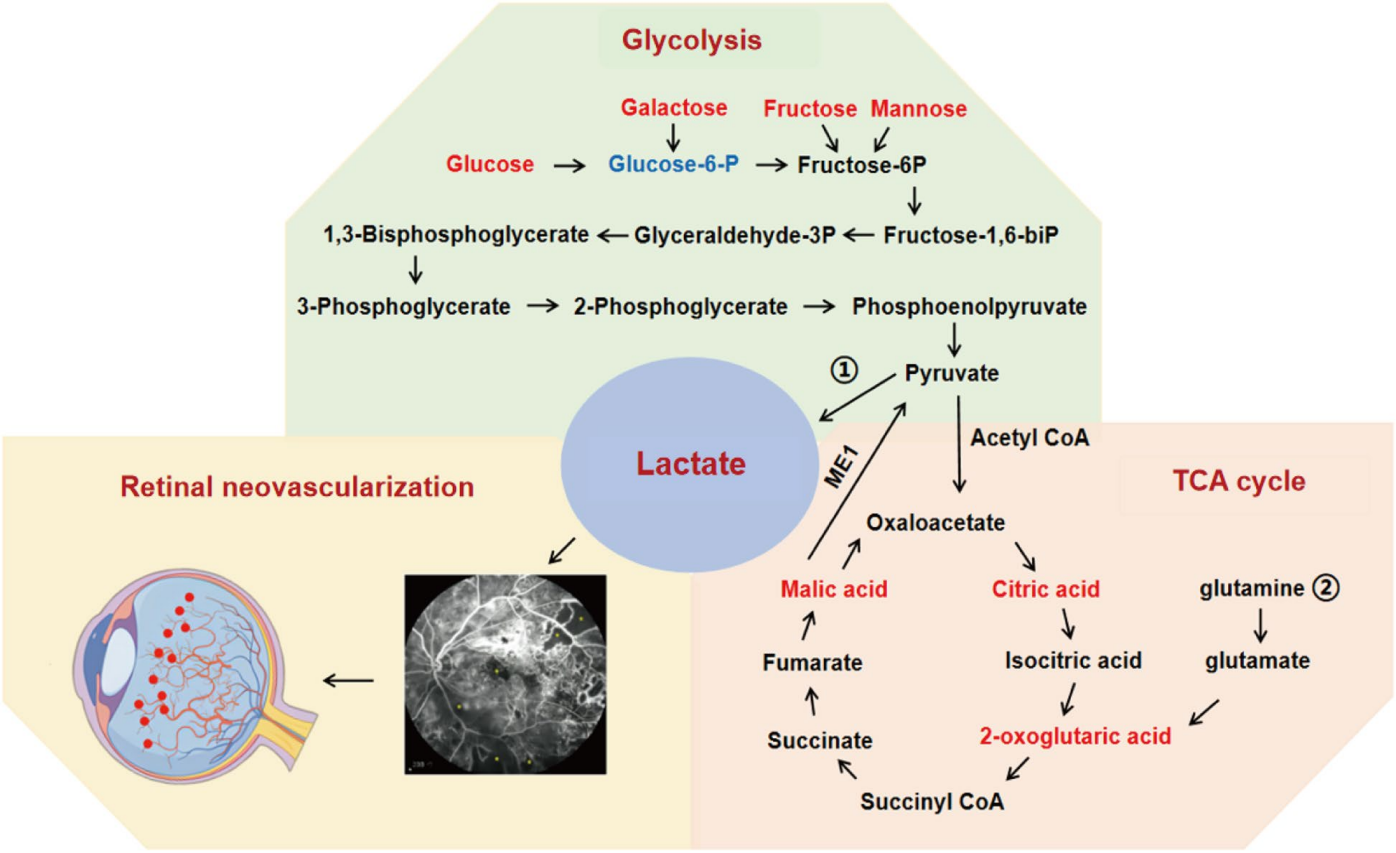
Diagram of lactate connected glycolysis and TCA cycle as well as the effect to promote retinal neovascularization. The lactate is generated through two principal metabolic pathways: ① glycolysis; ② glutamine metabolism. Red indicates the upregulation of glycometabolites, blue indicates the downregulation of glycometabolites. ME1: malic enzyme

The study revealed glycometabolic features in tear samples of PDR subjects. It is the the first time to identify the levels of fasting lactate and monosaccharides (D-glucose, D-fructose, D-galactose, and D-mannose) in the tear samples of the PDR subjects. Collectively, we found the tear lactate level of PDR subjects was the highest compared to other hexoses levels and suggested that monitoring lactate level in tears could be a non-invasive way to predict PDR **(**Fig. 6**)**.

Our untargeted metabolomics analysis revealed upregulation of the TCA cycle and D-glutamine/D-glutamate metabolism pathways in tear samples from PDR subjects. The results may be linked to the increase of lactate level. The conventional opinion conveyed that the glucose level was elevated and the retinal glycolysis was increased in DR progression [23, 24]. Due to insulin resistance, glucose is not fully utilized and converted to lactate to provide energy. Yet, lactate is currently considered as a signaling molecule to participate in other process apart from metabolism. The latest study indicated that the increase in oxidative phosphorylation mediated by lactate level leads to more pyruvate enter into the TCA cycle, which further increases ATP production. Lactate acts as a mitochondrial messenger to stimulate oxidative phosphorylation and activates the mitochondrial electron transport chain independently of its metabolism [25]. Similar study examined the fluxes of circulating metabolites in mice, and found that lactate is a primary source of carbon for the TCA cycle and thus of energy [26]. Except glycolysis pathway, lactate can be also generated through glutamine metabolism. Glutamate is initially metabolized to α-ketoglutarate and enters the TCA cycle. The glutamine-derived carbon is then incorporated into oxaloacetate, which is converted to malate and further decarboxylated to pyruvate. The final product of this pathway is lactate [27].

The levels of fasting lactate and monosaccharides (D-glucose, D-fructose, D-galactose, and D-mannose) in the tear samples of the PDR subjects were significantly higher than those of the non-diabetic subjects. The reason is that glucose produces lactate by anaerobic glycolysis when oxygen supply is insufficient or metabolic pathways are impaired during DR progression. Other monosaccharides (fructose, galactose, mannose) can be converted into phosphohexoses (glucose-6-phosphate, fructose-6-phosphate), which participates in glycolysis pathway [28]. Consistent results from other biofluids further support our findings. Previous study have reported elevated lactate levels in the vitreous humor of diabetic patients, particularly those with PDR [29]. Similarly, a large Chinese cohort study (n = 3933) found that lactate dehydrogenase (LDH) levels were associated with an increased prevalence of DR individuals [30]. Since tear lactate levels may fluctuate due to food intake, hydration status, environmental conditions, we collected fasting tear samples under standardized, quiet conditions. Additionally, all procedures were performed by the same operator to ensure consistency. Regarding collection methods, previous study found no significant differences in tear lactate levels between Schirmer’s test strips and micro-capillary tubes [31].

The ROC analysis can improve the fasting capillary glycemia for the screening of abnormalities in glucose tolerance [32]. Previous studies demonstrated that HbA1c or fasting blood glucose is effective in detecting diabetic retinopathy in different populations. A study from the United States reported that the AUC of HbA1c is a stronger discriminator of retinopathy (0.71) than fasting plasma glucose (0.65) [33]. On the contrary, in a Japanese study, the AUCs for fasting plasma glucose (0.668) and HbA1c (0.680) are very close [34]. A previous Chinese study indicated that the AUC for HbA1c is a weaker discriminator of retinopathy (81.4%) than fasting plasma glucose (83.7%) [35]. Consistent to the previous Chinese study, we, in the study, confirmed that fasting blood glucose showed high performance to distinguish DR from the non-diabetic subjects with an AUC of 0.871. Nevertheless, tear lactate could perform better to distinguish the PDR subjects from the NPDR subjects with an AUC of 0.896 than the other 4 monosaccharides in tears (AUC from 0.590 to 0.662) and the fasting blood glucose (AUC: 0.566). Altogether, tear lactate plays a leading role in the AUC for combination of 5 metabolites related to glycometabolism and could be a potential biomarker for the PDR evaluation.

We also further verified the potential effect of tear lactate and blood glucose level on RNV by retinal non-perfusion area in FFA. Several alterations of DR (loss of pericytes, thickening of endothelial cell basement membranes and microaneurysms) contribute to the breakdown of the blood-retinal barrier and result in abnormal retinal vasculatures. The vascular loss initially targets retinal capillary beds and may progressively extend to larger arterioles and veins, ultimately forming non-perfusion areas. These ischemic regions develop gradually and are clearly detectable via angiography, which further stimulates VEGF expression. Consequently, the extent of retinal non-perfusion correlates with DR severity and serves as a key indicator for RNV [36]. Similarly, retinal non-perfusion area was positively correlated with RNV in 47 quiescent PDR eyes [37]. Beyond stimulating RNV, lactate also promotes endothelial-cell activation and angiogenesis in other tissue or cells [38, 39]. Subsequently, choroid sprouting assay was conducted to further validate the effect of lactate on angiogenesis. Choroidal vasculopathy in DM mirrors vascular changes observed elsewhere in the eye and body. Given the critical role of choroid in nourishing and oxygenating the outer retina, these vascular alterations likely contribute significantly to diabetic retinopathy (DR) pathogenesis [40].

There were several limitations in this study. Firstly, the analyses were not stratified by age, gender, and disease severity due to a small number of study subjects and is absence of an independent validation cohort. This constraint may affect the generalizability of our findings across different patient subgroups. While our initial findings suggest tear lactate levels may serve as a potential marker for PDR, the predictive performance requires further evaluation in larger, multicenter validation studies. Furthermore, further investigation should be verified whether anti-VEGF treatment or other medications for DM affect tear lactate levels over time. Besides, the microbiomes on the ocular surface of the patients might also influence the sugar levels in the tear samples [41].

In summary, the study revealed the glycometabolic features in tears of PDR subjects and suggested that tear lactate could be a novel marker for the PDR evaluation.

## Materials and Methods

### Study subjects

The cohort is the same as our previous publication [16]. However, one PDR sample was excluded from the targeted metabolomics experiment due to insufficient tear volume. Finally, 19 type-2 DM patients with PDR, 21 type-2 DM patients with NPDR, and 21 non-diabetic subjects were enrolled. The detailed DR stages were based on Diabetic Retinopathy Preferred Practice Pattern in 2019 [42]. In brief, PDR diagnosis required presence of either neovascularization or vitreous/pre-retinal hemorrhage. NPDR staging adhered to criteria: mild (microaneurysms only), moderate (exceeding mild but not meeting severe criteria), or severe (fulfilling ≥ 1 of the 4–2-1 rule criteria: > 20 intraretinal hemorrhages per quadrant, venous beading in ≥ 2 quadrants, or prominent microvascular abnormalities in ≥ 1 quadrant), absent proliferative features. To minimize the inter-group difference, similar stage, disease duration and clinical manifestation of the patients were included in the NPDR study. The exclusion criteria comprised the participants with: (1) ocular surface disorders (e.g., conjunctivitis, meibomian gland dysfunction) or other fundus diseases (e.g., age-related macular degeneration); (2) or several systemic diseases (e.g., infections, cancers, immune disorders); (3) or prior ocular surgery; (4) or inability to cooperate with tear collection. All the study subjects were on fasting and did not use any eye drops in a period of 12 h. Approval for this research was obtained from the Human Medical Research Ethics Committee at Joint Shantou International Eye Center of Shantou University and the Chinese University of Hong Kong (approval number: EC20200609(6)-P24), following Declaration of Helsinki guidelines.

### Tear sample collection

All tear samples were collected by the Schirmer strips [43]. The patients were asked to look up and gently pulled down the lower eyelid. Then the strips were placed at the conjunctival sac 1/3 of the lower eyelid (avoid the cornea, close to the conjunctiva) for 10 min and gently removed by a sterile forceps. For each participant, one strip was collected from each eye, with bilateral samples subsequently pooled to create a single composite sample. All tear samples were frozen at −80 °C for and pre-processed prior to further analysis. Detailed methods have been described in our previous publication [16].

### Clinical data detection and metabolomics analysis

For each participant, age, gender, DM duration, DR duration for each subject were recorded. Fasting blood glucose level, HbA1c, fructosamine, triglycerides, total cholesterol, high density lipoprotein, low density lipoprotein, urea, and creatinine were measured by automatic biochemical analyzer (Fluorescence Spectrophotometer F-7100; Hitachi High-Tech Corporation, Tokyo, Japan). In DR subjects, the retinal non-perfusion areas were examined via FFA (Heidelberg Engineering GmbH, Heidelberg, Germany). In terms of the metabolomics research, all tear samples were assessed in the same single-run experiment. The procedures of untargeted metabolomics as been described in our previous study [16]. For targeted metabolomics, the levels of lactate, glucose, fructose, galactose and mannose in tears at fasting were determined by the LC–MS (Waters, UPLC; Thermo, Q Exactive)/MS platform (AB SCIEX 5500 QQQ -MS). The LC/MS analysis was conducted using an ACQUITY UPLC HSS T3 column (2.1 × 100 mm, 1.8 μm) maintained at 40 °C, with a mobile phase consisting of 0.01% formic acid in water (phase A) and acetonitrile (phase B) delivered at a flow rate of 0.3 mL/min and an injection volume of 6 μL. The mass spectrometry analysis employed electrospray ionization with the following optimized parameters: ionspray voltage = −4200 V, curtain gas = 20 arb, collision gas = 9 arb, ion source temperature = 450 °C, and ionsource gas = 35 arb.

### Choroid Sprouting Assay

C57BL/6 J mice (Vital River, China) around postnatal (P) 6 d were used in the study. Following euthanasia, the eyes were promptly enucleated and placed in PBS solution for further processing. The cornea and lens were removed from the anterior segment, and the sclera-choroid-RPE complex was dissected away from the retina. The isolated posterior tissues were then divided into 1 mm × 1 mm pieces. The dissected fragments were placed on 50 µL Matrigel (Corning, China) droplets in 24-well plates (Cellvis, USA), with all steps performed on ice to prevent Matrigel polymerization. Plates were then incubated at 37 °C for 30–60 min (without medium) to allow gel solidification. Subsequently, 500 µL of EGM-2 Basal Medium (Lonza, China) was added to each well, followed by incubation at 37 °C with 5% CO₂ for 48 h prior to treatments. For the experimental group, 1 mM lactate (matching tear lactate levels in PDR subjects of Fig. 2e) was supplemented into the EGM-2 medium. Daily photographs of individual explants were acquired over a 5-day period using a fluorescence microscope (Zeiss, Germany). Sprouting areas were quantified using ImageJ software. The study was approved by the Ethics Committee for Experimental Animal at the Joint Shantou International Eye Center of Shantou University and the Chinese University of Hong Kong (approval number: EC20200730(4)-P01).

### Statistical analysis

Continuous variables were expressed as mean ± standard deviation (SD) and analyzed using two-sample t-tests or one-way ANOVA with post-hoc false discovery rate (FDR) correction. Fisher’s exact test was applied for categorical variables when the number is less than 5. All *P* values were calculated after adjusting age. The multivariable logistic regression was fitted to evaluate the relationship between the tear lactate or monosaccharides and PDR. Pearson correlation analysis was applied for the correlation between tear lactate levels and monosaccharides levels or the retinal non-perfusion area. Receiver operating characteristic curve (ROC) analysis was performed to evaluate the predictive performance of the monosaccharides and lactate on DR as well as PDR. The retinal non-perfusion areas were calculated by Image J. All statistical analyses were performed using commercially available software (IBM SPSS Statistics 22; SPSS Inc., Chicago, IL) and GraphPad Prism 9.0 (GraphPad Software, San Diego, CA, USA). Statistical significance was considered as *P* < 0.05.

## Supplementary Information

Supplementary Material 1.

## Data Availability

The data supporting this study are available from the corresponding author upon reasonable request.
