# Supplementary material for: Tear lactate improves the evaluation of proliferative diabetic retinopathy in type-2 diabetes patients
Source: Mol Biomed. 2025 Jul 18;6:52. doi: 10.1186/s43556-025-00297-0 (PMC12270997; doi:10.1186/s43556-025-00297-0)
Supplement: Supplementary file 1 — Supplementary Material 1. [file 43556_2025_297_MOESM1_ESM.docx]

**Tear lactate improves the evaluation of proliferative diabetic retinopathy in type-2 diabetes patients**

Xin Wen ^1,2^, Tsz Kin Ng ^1,2,4^, Guihua Zhang ^1^, Haoyu Chen ^1^, Zhenggen Wu ^1^, Qingping Liu ^1,3*^, Mingzhi Zhang ^1,*^

^1^ Joint Shantou International Eye Center of Shantou University and the Chinese University of Hong Kong, Shantou, Guangdong, China.

^2^ Shantou University Medical College, Shantou, Guangdong, China.

^3^ Shantou University Medical College Eye Research Institute, Shantou, Guangdong, China.

^4^ Department of Ophthalmology and Visual Sciences, The Chinese University of Hong Kong, Hong Kong, China.

*** Corresponding author:**

**Qingping Liu, Ph.D.**

Joint Shantou International Eye Center of Shantou University and the Chinese University of Hong Kong, North Dongxia Road, Shantou, Guangdong, China 515041

Email: lqp@jsiec.org; Phone: +86-0754 88393560; Fax: +86-0754 88393560

**Mingzhi Zhang, M.D.**

Joint Shantou International Eye Center of Shantou University and the Chinese University of Hong Kong, North Dongxia Road, Shantou, Guangdong, China 515041

Email: zmz@jsiec.org; Phone: +86-0754 88393560; Fax: +86-0754 88393560

**Supplemental Table 1. Performance of tear monosaccharides, blood glucose as well as**

**combination of tear lactate and monosaccharides as a screening candidate for the evaluation of proliferative diabetic retinopathy**

| Metabolites | Groups | AUC (95% CI) | Cut-off value | Sensitivity | Specificity |
| --- | --- | --- | --- | --- | --- |
| Tear lactate | DR vs Control | 0.658 (0.519-0.797) | 786.91 μM | 0.762 | 0.550 |
|  | PDR vs NPDR | 0.896 (0.802-0.990) | **848.5 μM** | 0.905 | 0.737 |
| Tear glucose | DR vs Control | 0.709 (0.574-0.844) | 70.823 μM | 0.714 | 0.725 |
|  | PDR vs NPDR | 0.590 (0.403-0.777) | 97.686 μM | 0.714 | 0.579 |
| Tear fructose | DR vs Control | 0.737 (0.607-0.867) | 76.998 μM | 0.714 | 0.75 |
|  | PDR vs NPDR | 0.649 (0.470-0.828) | 107.25 μM | 0.810 | 0.579 |
| Tear galactose | DR vs Control | 0.738 (0.607-0.869) | 74.123 μM | 0.762 | 0.775 |
|  | PDR vs NPDR | 0.662 (0.484-0.839) | 103.14 μM | 0.810 | 0.632 |
| Tear mannose | DR vs Control | 0.727 (0.596-0.859) | 90.993 μM | 0.714 | 0.75 |
|  | PDR vs NPDR | 0.650 (0.471-0.830) | 128.12 μM | 0.810 | 0.579 |
| Blood glucose | DR vs Control | 0.871 (0.784-0.959) | 6.755 mM | 0.857 | 0.800 |
|  | PDR vs NPDR | 0.566 (0.372-0.761) | 10.82 mM | 0.905 | 0.421 |
| Combination of tear lactate and 4 monosaccharides | DR vs Control | 0.754 (0.628-0.879) | / | 0.810 | 0.675 |
|  | PDR vs NPDR | 0.947 (0.884-1.000) | / | 0.857 | 0.947 |

DR: diabetic retinopathy; NPDR:non-proliferative diabetic retinopathy; PDR:proliferative diabetic retinopathy
